# A Deep Learning Onion Peeling Approach to Measure Oral Epithelium Layer Number

**DOI:** 10.3390/cancers15153891

**Published:** 2023-07-31

**Authors:** Xinyi Zhang, Frederico O. Gleber-Netto, Shidan Wang, Kevin W. Jin, Donghan M. Yang, Ann M. Gillenwater, Jeffrey N. Myers, Renata Ferrarotto, Curtis R. Pickering, Guanghua Xiao

**Affiliations:** 1 Quantitative Biomedical Research Center, Peter O’Donnell Jr. School of Public Health, The University of Texas Southwestern Medical Center, Dallas, TX 75390, USAshidan.wang@utsouthwestern.edu (S.W.); donghan.yang@utsouthwestern.edu (D.M.Y.); 2 Department of Head & Neck Surgery, The University of Texas MD Anderson Cancer Center, Houston, TX 77030, USAagillenw@mdanderson.org (A.M.G.); jmyers@mdanderson.org (J.N.M.); 3 Department of Thoracic Head & Neck Medical Oncology, The University of Texas MD Anderson Cancer Center, Houston, TX 77030, USA; rferrarotto@mdanderson.org; 4 Department of Surgery, Yale School of Medicine, New Haven, CT 06510, USA; 5 Department of Bioinformatics, The University of Texas Southwestern Medical Center, Dallas, TX 76104, USA; 6 Simmons Comprehensive Cancer Center, The University of Texas Southwestern Medical Center, Dallas, TX 76104, USA

**Keywords:** oral cavity and pharyngeal cancer, oral epithelium, histology, medical image analysis, deep learning

## Abstract

**Simple Summary:**

Oral cavity and pharyngeal cancer affects 560,000 people worldwide on a yearly basis. Despite advances in treatment, the survival rate remains poor, while prognoses typically improve with early diagnosis. The epithelium of the oral cavity often exhibits abnormal cellular growth, or dysplasia, that predisposes the patient to cancer depending on severity. Our study aims to address the current lack of rigorous quantitative methods for analyzing histopathological features relevant to clinical diagnosis, such as cellular morphology and epithelial layer number. We developed a deep learning approach that segments the oral epithelium and counts epithelial layer number within H&E-stained whole slide images. Our results demonstrate the feasibility of this automated approach for segmenting oral epithelium and counting its layer number. We also show its clinical relevance by comparing oral epithelium layer numbers between dysplasia of different severities.

**Abstract:**

Head and neck squamous cell carcinoma (HNSCC), specifically in the oral cavity (oral squamous cell carcinoma, OSCC), is a common, complex cancer that significantly affects patients’ quality of life. Early diagnosis typically improves prognoses yet relies on pathologist examination of histology images that exhibit high inter- and intra-observer variation. The advent of deep learning has automated this analysis, notably with object segmentation. However, techniques for automated oral dysplasia diagnosis have been limited to shape or cell stain information, without addressing the diagnostic potential in counting the number of cell layers in the oral epithelium. Our study attempts to address this gap by combining the existing U-Net and HD-Staining architectures for segmenting the oral epithelium and introducing a novel algorithm that we call Onion Peeling for counting the epithelium layer number. Experimental results show a close correlation between our algorithmic and expert manual layer counts, demonstrating the feasibility of automated layer counting. We also show the clinical relevance of oral epithelial layer number to grading oral dysplasia severity through survival analysis. Overall, our study shows that automated counting of oral epithelium layers can represent a potential addition to the digital pathology toolbox. Model generalizability and accuracy could be improved further with a larger training dataset.

## 1. Introduction

Head and neck squamous cell carcinoma (HNSCC), specifically in the oral cavity as oral squamous cell carcinoma (OSCC), is one of the most common cancers in the world, posing a threat to human health and significantly affecting patients’ quality of life. Each year, there are an estimated 560,000 new cases of HNSCC worldwide [1]. Among them are 54,000 new cases in the United States [2], which include 38,800 males and 15,210 females. Roughly 10,850 people were expected to die of HNSCC per year [2], constituting 1.8% of all cancer deaths in America.

HNSCC is a complex disease with a wide range of patient outcomes depending on histological subtypes and tumor stages. Despite advances in treatment over the years, the 5-year survival rate of HNSCC patients remains low. Usually, early detection of cancer or precancerous changes lead to better patient prognosis. The epithelial lining of the oral cavity sometimes undergoes certain changes that place it at a higher risk of malignant transformation [3]. Such precursor lesions are named oral potentially malignant disorders (OPMD), which may contain epithelial dysplasia at the histological level. Evidence suggests that the more severe the dysplasia in a single lesion, the more likely it is to develop into cancer [4].

Dysplasia grading is vital to disease monitoring, where conventional qualitative histopathological evaluation based on light microscopic examination of hematoxylin and eosin-stained (H&E) slides is the standard method for assessing the malignant potential of lesions [4,5]. But due to a lack of established morphological criteria and the biological nature of dysplasia, the task is subjective and lacks intra- and inter-observer reproducibility [4,6]. There is no established quantitative technique by which histopathologically significant features of the diseased tissue, such as cell shape, cell density, and cell layer thickness, can be analyzed.

It follows that rigorously quantitative procedures that do not rely on human perception. Several studies have explored their potential. M. Abdel-Salam et al. used the Leitz TAS-Plus image analyzer (E. Leitz, Inc., Rockleigh, NJ, USA) in the early 1990s to assess 14 features of nuclei within oral epithelial lesions, including area, perimeter, maximum/minimum diameter, and so on [7]. Abu Eid et al. used traditional computer image analysis techniques, such as watershed segmentation, to quantify cell information in oral epithelial dysplasia biopsy samples in an attempt to classify the severity level of dysplastic cells [3]. However, these studies were limited by the technology available at the time with less accuracy. Moreover, several important features for oral dysplasia pathological diagnosis were not explored, such as cell layer-related features.

Thanks to the rapid development of deep learning image analysis, we are now able to accurately delineate the location of cells and tissues, precisely recognizing their exact cell type [8,9]. Deep learning segmentation algorithms are divided mainly into two categories: semantic and instance segmentation algorithms. The goal of semantic segmentation is to segment an image into categories with distinct meanings [10]. The most widely used semantic segmentation algorithm is the U-Net architecture, proposed by Ronneberger et al. [11]. On the other hand, the goal of instance segmentation is to segment different objects regardless of whether they belong to the same category when close to each other. In contrast to semantic segmentation, instance segmentation aims to detect the boundaries of each object, whereas the former merely segments the combined area of these objects [10]. Within instance segmentation, the Mask R-CNN model achieves high performance [12], has attracted great attention, and is widely adopted. Based on the Mask R-CNN architecture, our lab developed the HD-Staining model, which has achieved success in pathological image analysis [8].

In order to improve diagnostic accuracy and consistency, it is essential to develop a more advanced deep learning-based computer-aided strategy to assist dysplasia pathology diagnosis. Previous studies have mostly been limited to deriving shape information from single nuclei or cell stain information [8,10]. There has been no research on the automated analysis of epithelial layer structure. Most dysplastic tissue exhibits altered epithelial stratification, much of it hyperplastic, meaning that there are more layers of epithelial cells. In current clinical practice, although layer alteration is a consideration in grading oral dysplasia, the process depends on the pathologist’s experience and observational skills. There is currently no established method to quantify cell layer number, which could represent a substantial addition to the pathologist’s toolbox. This calls for several automation steps: firstly, segmenting the epithelium area as well as the epithelial cells within it, and secondly, a counting algorithm to count the layers.

**Figure 1:**
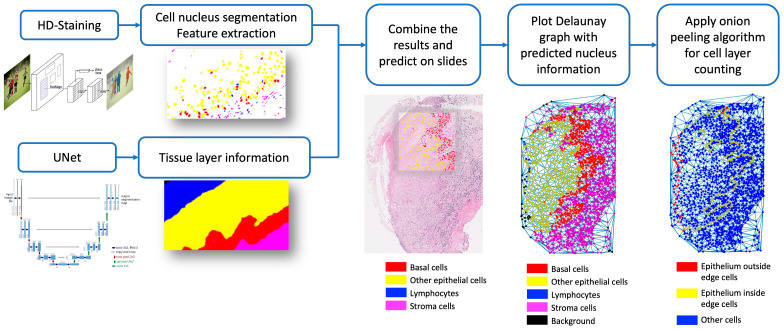
Overview of study design.

In this study, we developed a computational method that quantifies cell layer number within the oral epithelium. We first applied U-Net to delineate the oral epithelial tissue as well as subdivision of the basal cell layer within the epithelium; then, we used the HD-Staining model for nuclei segmentation. The results of the two models were combined to achieve satisfactory nuclei segmentation and classification. Subsequently, we developed a special algorithm to count the cell layers, which we call Onion Peeling. The algorithm-produced cell layer number was compared with an independent, manually counted number, producing a correlation of 0.849. Median cell layer counts were significantly different among different severity groups of oral dysplasia; severe dysplasia had the highest counts, which is consistent with the pathological diagnosis criteria. In addition, survival analysis showed that patients with a lower cell layer count were associated with a better prognosis. Our workflow is depicted in Figure 1.

## 2. Materials and Methods

### 2.1. Dataset

H&E-stained histology whole slide images (WSIs) and corresponding clinical information were collected from two independent datasets.

The first dataset comprised 13 oral mucosa H&E slides with normal oral epithelium retrieved from the archives of the Oral Pathology Service of the School of Dentistry at Universidade Federal de Minas Gerais in Brazil (Supplementary Table S1). This cohort included biopsies of clinically normal mucosa and oral diseases associated with changes in the lamina propria while maintaining normal structure of the oral epithelium. 

The other dataset, which contained OPMD cases, was retrieved from oral premalignancy cases that were part of the EPOC trial at The University of Texas MD Anderson Cancer Center. This cohort included 135 patients with corresponding slides, including both cancer and non-cancer patients. The slides had epithelial lesions at risk for future progression to cancer. 

An expert pathologist (Dr. Frederico O. Gleber-Netto) reviewed all slides to confirm the diagnoses and circled regions of interest (ROI) for analysis. Pathologist F. O. G.’s diagnosis was considered the golden standard in this study.

### 2.2. U-Net Model Training

#### 2.2.1. Preparation of Training, Validation, and Testing Sets

Image patches were extracted from areas within and around the oral epithelium on 18 OPMD slides. A total of 168 image patches for training were selected from 12 slide images and 60 for validation were selected from 6 slide images. Patches of size 500 × 500 pixels at 10× magnification were randomly taken from the ROIs on the slides. They were then manually examined to avoid markers and eliminate similar images taken at the same spot. The ground truth masks were then manually labeled in Adobe Photoshop. All pixels within an image patch were labeled as one of the following: epithelium basal layer, epithelium other, stratum corneum, non-epithelium tissue, background.

#### 2.2.2. Model Training

MobileNetV2 [13], which utilizes U-Net as its underlying architecture, was selected as our network backbone. U-Net, so named because of its near-symmetrical shape, is a precise and extensible convolutional network specifically developed for image segmentation. It consists of a “contracting” path that performs much like a typical convolution network and an “expansive” path that learns localized classification contexts. U-Net’s architecture enables it to produce detailed segmentation maps from limited training samples, which is of particular importance to applications in biomedical image analysis [11,14]. To ensure model robustness to changes in staining conditions and tissue distortions, we used random projective transformations, color shifting for the whole image, color shifting and variation for each color channel, and random horizontal and vertical flipping in data augmentation. To ensure independence between the training and validation datasets, image patches from the same slide were assigned together.

For the training process, we used the Dice coefficient to observe the training process. The batch size was set to 4, the learning rate was initially set to 0.001 and optimized by the Adam optimizer, and the maximum number of training epochs was set to 100. The model achieved the lowest loss on the validation set at the 89th epoch. This model was selected and used in the following analysis to avoid overfitting. Python (version 3.6.4) and Python libraries (Keras, version 2.2.4; openslide-python, version 1.1.1; and tensorflow-gpu, version 1.13.1) were used.

### 2.3. HD-Staining Model Training

#### 2.3.1. Dataset

A total of 13 normal epithelial slides and 17 OPMD slides were used to train the HD-Staining model. Image patches of size 500 × 500 at 40× magnification were randomly taken from the ROIs around the oral epithelium. Photoshop software (version 24.7) was used to label the ground truth masks. In these patches, four different types of cell nuclei were labeled: all pixels with basal cell nuclei, lymphocyte nuclei, other epithelial nuclei, and stroma nuclei were labeled according to their categories and all remaining pixels were considered as “other”. These labels were then used as the ground truth to train the HD-Staining model. The labeled images were randomly divided on a slide level into training, validation, and testing sets to ensure independence among these datasets. There were 184 images for training, 45 for validation, and 24 for the testing dataset.

#### 2.3.2. Model Training

The HD-Staining model architecture is based on that of Mask R-CNN, a simple and generalizable convolutional network for pixel-level object instance segmentation but adapted for pathology image analysis with customized data loading, image augmentation, image centering, and scaling processes [12]. In their respective studies, Mask R-CNN was trained on the COCO dataset, and HD-Staining was fine-tuned on lung adenocarcinoma pathology images from 135 patients in the National Lung Screening Trial, as previously described in a paper from our lab [8]. For our study, we further fine-tuned a Keras implementation of HD-Staining on our OPMD training dataset. Images were standardized (centered and scaled to have zero mean and unit variance) for each RGB channel. The same data augmentation technique as described in Section 2.2.2 was applied to increase model generalizability. Python (version 3.6.4) and Python libraries (Keras, version 2.2.4; openslide-python, version 1.1.1; and tensorflow-gpu, version 1.13.1) were used.

### 2.4. Combined Model Prediction on Whole Slides

After establishing the U-Net and HD-Staining models for oral epithelium tissue, we combined the two models to predict on WSIs of the OPMD dataset. Specifically, a 500 × 500 image at 10× magnification would be drawn from the slide for U-Net prediction, and at the same location, the corresponding 2000 × 2000 image at 40× magnification was drawn for the HD-Staining model. Then, the U-Net prediction result was generated and saved. The 2000 × 2000 image at 40× magnification was divided into partially overlapping 500 × 500 images to be processed by the HD-Staining model. The edges of the smaller image predictions were discarded to minimalize the edge effect.

**Figure 2:**
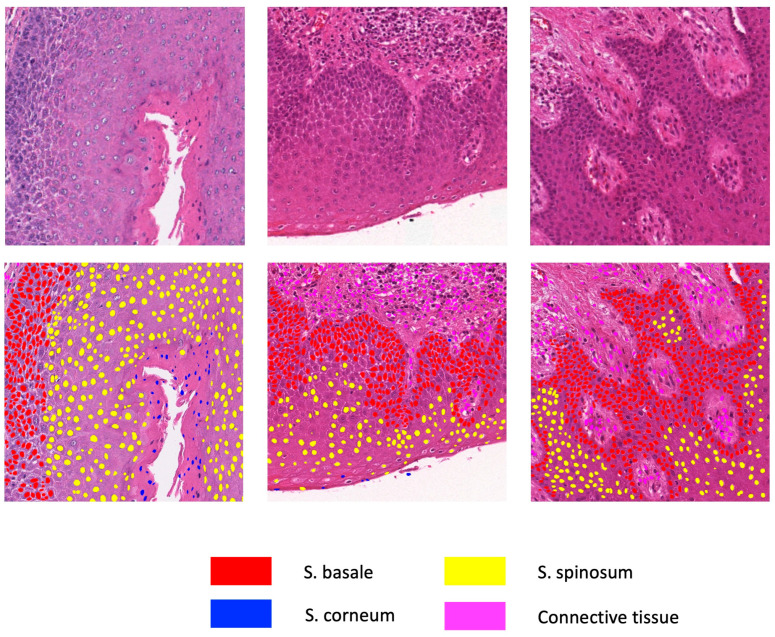
Image patch prediction results combining HD-Staining and U-Net models.

To combine results from both models, any prediction of individual cells from the HD-Staining model would have its center location checked against the result from the U-Net model. The final classification of the cell category was based on U-Net, while maintaining the segmentation from HD-Staining. Sample results are shown in Figure 2. WSIs were predicted by predicting patch to patch as described above, generating a spreadsheet of nuclei information of all the cells in the image.

### 2.5. Onion Peeling Algorithm

#### Process Description

**Figure 3:**
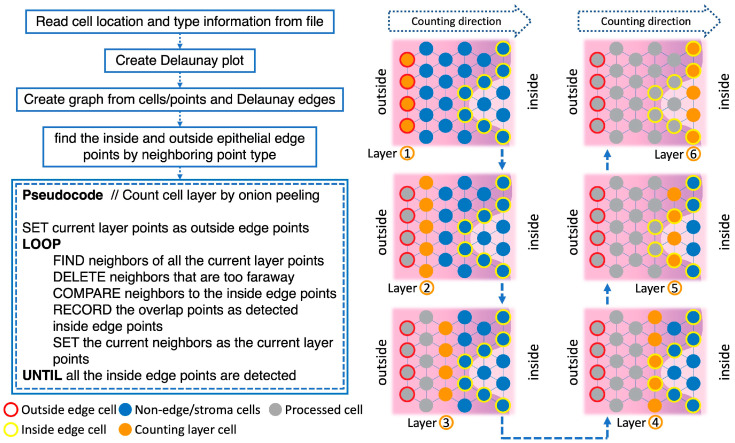
Pseudocode for Onion Peeling algorithm and illustration of the process.

Figure 3 displays the pseudocode and an illustration of the Onion Peeling algorithm. First, the algorithm reads cell location and type information from spreadsheet files. This information was used to generate a Delaunay plot representing the distribution of the cells, where all line-connected points are considered as neighboring cells. To achieve our goal of layer counting, a graph based on the cell location and type information, as well as Delaunay plot edge information was generated.

A pivotal task in this process was locating the inside and outside nuclei of the epithelium, which formed the basis of our counting task. We distinguished the inside and outside epithelial edge points by neighboring point type. This was achieved by first locating all neighbors of one nucleus. If there were background or corneum cells among its neighbors, then it was an outside edge point; if there were stroma cells among its neighbors, then it was an inside edge point.

After finding epithelial edge points, we also located several small, disconnected areas. These either resulted from small connective cell areas in the epithelium or imperfections in the tissue such as cracks. Filtering these out of the edge points set was important to correctly count the epithelial layer number. This was conducted by separating the connected components of the graph and counting the number of cells in each connected component; if the number of cells in connected components is less than 20, then these cells were re-categorized as “non-edge” cells.

After determining the edge points of the epithelium, we executed the Onion Peeling algorithm. The algorithm began from the inside (basal) layer to the outside edge points, marking them as the current layer, and then located the neighbors of all current layer points, deleting neighbors that were too far away (more than 500 pixels apart). The detected neighbors were compared with the inside edge points to check for any overlap. After comparison, the current layer number was recorded, as well as the number of overlapping points. The neighbors were set as the new current layer, and the previous steps repeated until all inside edge points were detected.

### 2.6. Statistical Analysis

ANOVA F-tests were used to compare the median layer number distribution among different dysplasia disease stages, including hyperkeratosis/hyperplasia (*n* = 45), mild/moderate dysplasia (*n* = 82), and severe dysplasia/carcinoma (*n* = 8).

Kaplan–Meier plots were used to summarize the progression-free survival curves of the patients in predicted low- or high-risk groups. A multivariate Cox proportional hazard (CoxPH) model was used to study the association between the average epithelium layer number and patient survival outcome and to calculate the hazard ration (HR). 

All survival analyses were performed in R (version 4.0.3). The R package “survival” was used. The results were considered significant if the resulting two-tailed p-value was less than 0.05.

## 3. Results

### 3.1. Model Training

#### 3.1.1. U-Net Model

The U-Net model was trained for 100 epochs. After comparison of the mean intersection over union (IoU) in the validation dataset, the 89th epoch was selected as the final U-Net model for following study development. The validation set mean IoU was 0.70, and the validation weighted accuracy was 0.92. The tissues on slides were segmented and classified into four categories: basal cell nuclei, lymphocyte nuclei, other epithelial nuclei, and stroma nuclei, while all other tissues and space were considered background. An example segmentation result is shown in Figure 2.

#### 3.1.2. HD-Staining Model

The developed HD-Staining model segments and classifies individual nuclei. The segmented cell nuclei were classified into four categories: basal cell nuclei, lymphocyte nuclei, other epithelial nuclei, and stroma nuclei, while all other tissues and space were considered background. The validation set coverage (detected cells with a mean IoU over 0.5) was 0.73, and the mean IoU was 0.76.

#### 3.1.3. Combining U-Net and HD-Staining

We accepted the instance segmentation results from HD-Staining and classified them according to the prediction results from U-Net. Figure 2 shows several examples of the predicted image patches. The combined model was then applied to the whole slide, except for areas that a pathologist deemed should be excluded. All information of the detected cell nuclei, including cell location on the slide, size, and predicted cell type, was recorded and saved.

### 3.2. Onion Peeling

**Figure 4:**
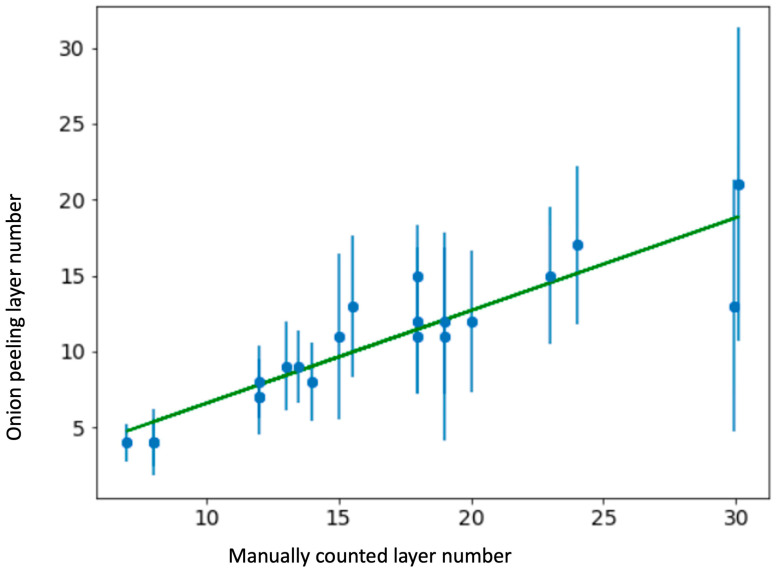
Onion Peeling results (at 0.5 quantile, *n* = 20) compared to manual count. Coefficient of determination R^2^ = 0.849. Blue vertical lines are error bars of standard deviation of Onion Peeling layer counting at all locations of the inner epithelium edge of that slide.

A Delaunay triangle graph was constructed for each slide and the Onion Peeling algorithm was used to count the epithelium layer number. The layer number at each location of the inner epithelium edge was recorded. The median of all the layer numbers on one slide was selected as the thickness of that epithelium tissue. For a small subset (*n* = 20) of slides that we received early, manual layer number counting was conducted beforehand for comparison with the algorithm counting results. A comparison scatter plot is shown in Figure 4. Linear regression analysis showed a coefficient of determination (R^2^) of 0.849, which is satisfactory. The Onion Peeling algorithm was applied to all 135 slide images.

### 3.3. Statistical Analysis

#### 3.3.1. Analysis of Layer Number between Diagnosis Groups

**Figure 5:**
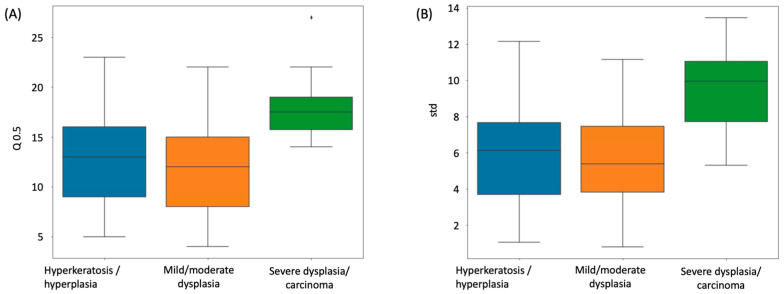
Distribution of median layer number of different dysplasia disease stages, comparing hyperkeratosis/hyperplasia (*n* = 45), mild/moderate dysplasia (*n* = 82), and severe dysplasia/carcinoma (*n* = 8). (**A**) Comparison of median epithelium layer number, ANOVA F-test *p*-value = 0.0012; (**B**) comparison of layer number standard deviation, ANOVA F-test *p*-value = 0.0006.

The median layer number from the Onion Peeling algorithm counting results was compared between different dysplasia disease stages. These disease stages were the diagnosis made by pathologists, including hyperkeratosis/hyperplasia (*n* = 45), mild/moderate dysplasia (*n* = 82), and severe dysplasia/ carcinoma (*n* = 8). Figure 5A shows a comparison of median epithelium layer number among these three groups; an ANOVA F-test resulted in a *p*-value of 0.0012. Furthermore, as shown in Figure 5B, we compared the standard deviation of the layer number of each slide, which is an indicator of the layer thickness variability of the epithelium; an ANOVA F-test showed a *p*-value of 0.0006. Of these two analyses, severe dysplasia/carcinoma had a significantly higher layer number and variation than the other two groups.

#### 3.3.2. Survival Analysis

**Figure 6:**
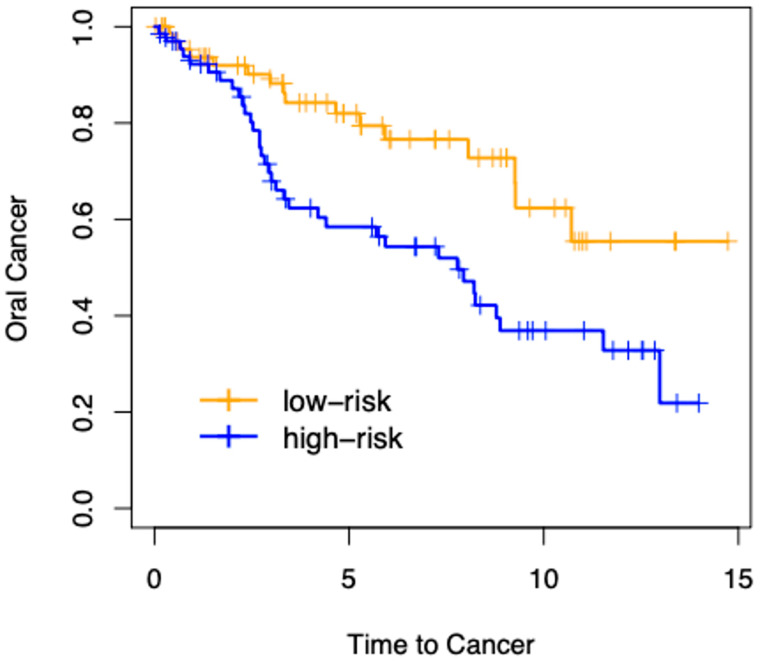
Kaplan–Meier plot showing survival difference between high layer number patients (high-risk) and low layer number patients (low-risk). A total of 135 patients were separated equally into two groups according to their epithelium layer number. Difference between the two risk groups: likelihood ratio test, *p* = 0.004; Cox proportional hazards model, *p* = 0.006.

We used the patient progression-free interval (PFI) as survival data to perform the following analysis. Figure 6 displays a Kaplan–Meier plot, which shows the survival difference between high layer number patients (high-risk) and low layer number patients (low-risk). A total of 135 patients were separated equally into two groups (*n* = 67 and *n* = 68, respectively) according to their epithelium layer number. The difference between the two risk groups was as follows: likelihood ratio test, *p*-value = 0.004; Cox proportional hazards model, *p*-value = 0.006.

## 4. Discussion

We presented a deep learning approach for determining the number of cell layers in the oral epithelium. We began by using U-Net to recognize and subdivide the oral epithelial tissue, then employing HD-Staining to segment cell nuclei, and finally merging the results of the two models. We developed an algorithm to count the cell layers automatically that we call Onion Peeling. The counted cell layer number was compared to an independent manual count, achieving a correlation of 0.849. The median cell layer counts of different severity groups of oral dysplasia varied significantly, with severe dysplasia having the greatest numbers, which is compatible with pathological diagnostic criteria. A preliminary survival analysis was conducted for our dataset of 135 oral dysplasia patients. After equally dividing the patients into two risk groups according to median cell layer counts, a significant difference (Cox proportional hazards model, *p*-value = 0.006) in survival curves between the groups demonstrated the value of the Onion Peeling algorithm in oral dysplasia grading.

Though our work has proven the concept and shown the value of counting epithelium cell layers, there is room for improvement. Firstly, manual counting is not a robust standard for comparison to the algorithmic results. Methods of counting layers may differ between individuals, and consistency among different human observers is poor, weakening the reliability of comparison between manual and algorithmic counts. Additionally, layer number is one of many aspects in dysplasia severity grading. Future research should include and quantify more aspects of the oral epithelium, such as nuclei shape and stain information. An expanded set of quantifiable cell and tissue parameters, combined with other clinical information, would make it easier for pathologists to accurately classify oral dysplasia lesions.

## 5. Conclusions

This paper demonstrates the feasibility of a deep learning approach to count oral epithelium cell layers, utilizing a combined model to segment the oral epithelial tissue and introducing a novel algorithm, Onion Peeling, to count the layers. Our experimental results confirm that Onion Peeling achieves high consistency and accuracy. In addition, we show the prognostic value of Onion Peeling in oral dysplasia severity grading. Further improvements could increase the algorithm’s accuracy and generalizability as a pathological classification tool.

## Data Availability

The data presented in this study are available in this article.

## Supplementary Materials

The following supporting information can be downloaded at https://www.mdpi.com/article/10.3390/cancers15153891/s1, Supplementary Table S1: Diagnosis of oral mucosa H&E slides with normal oral epithelium.

## References

[1] Sung H., Ferlay J., Siegel R.L., Laversanne M., Soerjomataram I., Jemal A., Bray F. (2021). Global Cancer Statistics 2020: GLOBOCAN Estimates of Incidence and Mortality Worldwide for 36 Cancers in 185 Countries. CA Cancer J. Clin..

[2] Siegel R.L., Miller K.D., Fuchs H.E., Jemal A. (2021). Cancer Statistics, 2021. CA Cancer J. Clin..

[3] Abu Eid R., Landini G. Oral Epithelial Dysplasia: Can Quantifiable Morphological Features Help in the Grading Dilemma?. Proceedings of the 1st ImageJ User and Developer Conference.

[4] Fleskens S., Slootweg P. (2009). Grading Systems in Head and Neck Dysplasia: Their Prognostic Value, Weaknesses and Utility. Head Neck Oncol..

[5] Warnakulasuriya S., Reibel J., Bouquot J., Dabelsteen E. (2008). Oral Epithelial Dysplasia Classification Systems: Predictive Value, Utility, Weaknesses and Scope for Improvement. J. Oral Pathol. Med..

[6] Krishnan L., Karpagaselvi K., Kumarswamy J., Sudheendra U., Santosh K., Patil A. (2016). Inter- and Intra-Observer Variability in Three Grading Systems for Oral Epithelial Dysplasia. J. Oral Maxillofac. Pathol..

[7] Abdel-Salam M., Mayall B.H., Chew K., Silverman S., Greenspan J.S. (1990). Which Oral White Lesions Will Become Malignant? An Image Cytometric Study. Oral Surg. Oral Med. Oral Pathol..

[8] Wang S., Rong R., Yang D.M., Fujimoto J., Yan S., Cai L., Yang L., Luo D., Behrens C., Parra E.R. (2020). Computational Staining of Pathology Images to Study the Tumor Microenvironment in Lung Cancer. Cancer Res..

[9] Chen Y., Li T., Zhang Q., Mao W., Guan N., Tian M., Yu H., Zhuo C. (2022). ANT-UNet: Accurate and Noise-Tolerant Segmentation for Pathology Image Processing. J. Emerg. Technol. Comput. Syst..

[10] Wang S., Yang D.M., Rong R., Zhan X., Xiao G. (2019). Pathology Image Analysis Using Segmentation Deep Learning Algorithms. Am. J. Pathol..

[11] Ronneberger O., Fischer P., Brox T., Navab N., Hornegger J., Wells W.M., Frangi A.F. (2015). U-Net: Convolutional Networks for Biomedical Image Segmentation. Medical Image Computing and Computer-Assisted Intervention—MICCAI 2015, Proceedings of the 18th International Conference on Medical Image Computing and Computer-Assisted Intervention, Munich, Germany, 5–9 October 2015.

[12] He K., Gkioxari G., Dollar P., Girshick R. Mask R-CNN. Proceedings of the IEEE International Conference on Computer Vision (ICCV).

[13] Sandler M., Howard A., Zhu M., Zhmoginov A., Chen L.-C. MobileNetV2: Inverted Residuals and Linear Bottlenecks. Proceedings of the IEEE Conference on Computer Vision and Pattern Recognition (CVPR).

[14] Siddique N., Paheding S., Elkin C.P., Devabhaktuni V. (2021). U-Net and Its Variants for Medical Image Segmentation: A Review of Theory and Applications. IEEE Access.

