# Supplementary material for: A Deep Learning Onion Peeling Approach to Measure Oral Epithelium Layer Number
Source: Cancers (Basel). 2023 Jul 31;15(15):3891. doi: 10.3390/cancers15153891 (PMC10416878; doi:10.3390/cancers15153891)
Supplement: Supplementary file 1 [file cancers-15-03891-s001.zip › cancers-2416387-supplementary.pdf]

Supplementary Table S1. Diagnosis of oral mucosa H&E slides with normal oral epithelium.

Sample	Group	Diagnosis	Epithelial status	Connective tissue status
1	Connective tissue disease - not cancer related	Fibrous hyperplasia	Normal	Altered
2		Fibrous hyperplasia	Normal	Altered
3		Fibrous hyperplasia	Normal	Altered
4		Intramuscular lipoma	Normal	Altered
5		Oral mucosa and Mucocele	Normal	Altered
6		Peripheral ossifying fibroma	Normal	Altered
7	Normal mucosa	Normal mucosa	Normal	Normal
8		Normal mucosa and sialoadenitis	Normal	Altered
9		Normal mucosa fragment	Normal	Normal
10		Normal mucosa with focal inflammatory infiltrate	Normal	Altered
11		Normal mucosa with inflammatory infiltrate	Normal	Altered
12		Oral mucosa and Ranula	Normal	Normal
13		Oral mucosa with nonspecific chronic inflammatory process	Normal	Altered
